# Recognition of Forward Head Posture Through 3D Human Pose Estimation With a Graph Convolutional Network: Development and Feasibility Study

**DOI:** 10.2196/55476

**Published:** 2024-08-26

**Authors:** Haedeun Lee, Bumjo Oh, Seung-Chan Kim

**Affiliations:** 1 Machine Learning Systems Laboratory School of Sports Science Sungkyunkwan University Suwon, Gyunggi-do Republic of Korea; 2 Department of Family Medicine SMG-SNU (Seoul Metropolitan Government - Seoul National University) Boramae Medical Center Seoul Republic of Korea; 3 Department of Family Medicine Seoul National University College of Medicine Seoul Republic of Korea

**Keywords:** posture correction, injury prediction, human pose estimation, forward head posture, machine learning, graph convolutional networks, posture, graph neural network, graph, pose, postural, deep learning, neural network, neural networks, upper, algorithms

## Abstract

**Background:**

Prolonged improper posture can lead to forward head posture (FHP), causing headaches, impaired respiratory function, and fatigue. This is especially relevant in sedentary scenarios, where individuals often maintain static postures for extended periods—a significant part of daily life for many. The development of a system capable of detecting FHP is crucial, as it would not only alert users to correct their posture but also serve the broader goal of contributing to public health by preventing the progression of chronic injuries associated with this condition. However, despite significant advancements in estimating human poses from standard 2D images, most computational pose models do not include measurements of the craniovertebral angle, which involves the C7 vertebra, crucial for diagnosing FHP.

**Objective:**

Accurate diagnosis of FHP typically requires dedicated devices, such as clinical postural assessments or specialized imaging equipment, but their use is impractical for continuous, real-time monitoring in everyday settings. Therefore, developing an accessible, efficient method for regular posture assessment that can be easily integrated into daily activities, providing real-time feedback, and promoting corrective action, is necessary.

**Methods:**

The system sequentially estimates 2D and 3D human anatomical key points from a provided 2D image, using the Detectron2D and VideoPose3D algorithms, respectively. It then uses a graph convolutional network (GCN), explicitly crafted to analyze the spatial configuration and alignment of the upper body’s anatomical key points in 3D space. This GCN aims to implicitly learn the intricate relationship between the estimated 3D key points and the correct posture, specifically to identify FHP.

**Results:**

The test accuracy was 78.27% when inputs included all joints corresponding to the upper body key points. The GCN model demonstrated slightly superior balanced performance across classes with an *F*_1_-score (macro) of 77.54%, compared to the baseline feedforward neural network (FFNN) model’s 75.88%. Specifically, the GCN model showed a more balanced precision and recall between the classes, suggesting its potential for better generalization in FHP detection across diverse postures. Meanwhile, the baseline FFNN model demonstrates a higher precision for FHP cases but at the cost of lower recall, indicating that while it is more accurate in confirming FHP when detected, it misses a significant number of actual FHP instances. This assertion is further substantiated by the examination of the latent feature space using t-distributed stochastic neighbor embedding, where the GCN model presented an isotropic distribution, unlike the FFNN model, which showed an anisotropic distribution.

**Conclusions:**

Based on 2D image input using 3D human pose estimation joint inputs, it was found that it is possible to learn FHP-related features using the proposed GCN-based network to develop a posture correction system. We conclude the paper by addressing the limitations of our current system and proposing potential avenues for future work in this area.

## Introduction

Improper posture is not merely a cosmetic concern but a health issue that can precipitate chronic conditions, significantly diminishing quality of life. Additionally, the treatment necessitates consistent rehabilitation exercises, leading to significant commitments in terms of time and financial resources. Forward head posture (FHP), characterized by the head jutting forward and shoulders rounding, is a prevalent consequence of modern lifestyles that involve extended periods of using laptops, mobile devices, or driving. Achieving optimal posture—where the spine maintains a neutral alignment with the gravitational line passing through the acromion and tragus—ensures stability and good postural health [1]. Deviations from this alignment, as seen in FHP, can place undue stress on the cervical spine. This can lead to persistent neck pain and headaches [2] and may escalate to issues such as gait disturbances, chronic fatigue, digestive disorders, and lumbar disk herniation [3,4]. FHP is also associated with increased activation in the thoracic and salivary muscles, angular and transcriptional changes in muscles, and a decrease in forced lung capacity [5]. Furthermore, it is linked to cervical radiculopathy, cervicogenic headaches, and dizziness [6-8].

Given the severity and widespread prevalence of FHP, the absence of an effective, robust posture monitoring system for various real-world conditions is alarming [9]. This emphasizes the necessity for preventative strategies, highlighting the urgency in creating a system capable of detecting FHP, correcting improper posture, and ultimately preventing the onset and progression of chronic conditions. Efforts to prevent FHP have included discussions on reliable and accessible measuring equipment and methods for assessing body posture. Researchers have explored various approaches such as radiographic image analysis [10] and physical measurements with medical instruments [11-13]. More specifically, a recent study introduced a novel wearable device to measure FHP, using a magnetometer and a permanent magnet for precise head posture calibration, which, when combined with accelerometer data and processed through machine learning algorithms, demonstrates high accuracy in assessing neck angles and determining FHP risk levels [12]. In their follow-up work, another recent study introduced a method for simultaneous detection of common posture issues, including FHP, using a novel combination of sensors and deep learning algorithms, achieving high accuracy in classifying these postural disorders [13]. In a recent study, researchers introduced a convolutional neural network system that demonstrated high accuracy in automated identification of anatomical landmarks in petrous temporal bone cone-beam computed tomography scans, achieving 0.958 in axial slices and 0.924 in coronal slices with statistical significance (*P*<.001) [14]. While these methods have proven reliable, they are limited by the need for specialized medical equipment and expert involvement, making them impractical for routine detection of injury risk in everyday life.

Meanwhile, advancements in computer vision technologies have significantly enhanced capabilities in real-time image processing and 3D human pose estimation from single images [15]. Leveraging this technological progress, we aim to propose a digital health care system designed to prevent chronic neck injuries by detecting FHP through advanced 3D human pose estimation techniques.

Our system initiates with a preprocessing stage in which a 2D red, green, and blue (RGB) image is input and analyzed to infer the corresponding 3D human poses via the VideoPose3D algorithm [16]. Central to the prediction process is a deep neural network tailored to harness the 3D joint coordinates along with their interconnectivity to accurately identify FHP.

To assess the effectiveness of our method, we conducted a series of experiments using a public data set containing 2D images depicting users in various sitting postures. The outcomes of these experiments confirm the viability of our approach, illustrating that a single 2D image can provide significant insights into a user’s posture by estimating their 3D pose. The primary contributions of our work are 2-fold: first, we formulated an FHP detection system using recent computational techniques and developed a graph convolutional network (GCN)–based robust algorithm specifically designed to recognize FHP from 3D human posture estimated from 2D images. Second, through our comprehensive experimental validation, we established the reliability and accuracy of our method in a real-world context.

The implications of our approach extend beyond individual health benefits to broader public health advancements. By enabling early detection and intervention for FHP with our proposed system, we contribute to alleviating a widespread public health concern. This establishes our system not only as a tool for personal health management but also as a valuable component in efforts to enhance public health impacts related to posture-induced conditions.

## Methods

### Ethical Considerations

This study uses publicly available data sourced from the StateFarm data set [17], the “Don’t be a Turtle” project [18], and various digital photos and videos from platforms such as YouTube. The use of human photography in this research was approved by the institutional review board of Sungkyunkwan University (SKKU 2023-03-011). No identifiable personal data were used in this study.

### Proposed Approach

#### Overview

**Figure 1:**
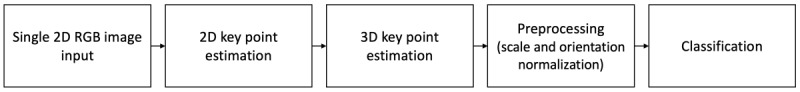
The base pipeline of the proposed method. The system first estimates the 2D key points, followed by the estimation of the 3D key points. These key points are then preprocessed by normalizing their scale and orientation. Finally, the normalized key points are used for classifying the posture to determine the presence of a forward head posture.

This paper presents a system designed to detect FHP using advanced deep learning techniques, aiming to enable early identification and management of postural irregularities, contributing to enhanced overall posture health and the prevention of related chronic conditions. In pursuit of this, we used Detectron2 [19] and VideoPose3D [16] for estimating 3D skeletal data from single 2D image inputs. Following this, the acquired data underwent preprocessing to enhance the classification accuracy of our deep-learning model. Figure 1 illustrates the foundational pipeline of our proposed method, which starts with a single RGB image input, followed by successive 2D and 3D key point estimation and preprocessing steps, and concludes with the classification phase. During the training phase, the data set was used for supervised learning to train the deep learning model.

#### Definition of FHP

In this paper, the definition of factors contributing to the risk of FHP is established based on multiple previous studies [3,10,12,20-32]. FHP is defined as a condition where the tragus is positioned anterior to the acromion. Conversely, a normal posture is characterized by the tragus being nearly aligned with the acromion. We established these criteria and had them cross-validated by 2 physical therapists (Bo Yeon Park and Dong Hyeon Park, from the Department of Physical Therapy, Namseoul University, Cheonan, Korea).

Throughout this paper, postures with a higher risk of leading to FHP are categorized as “FHP,” while those with a lower risk are categorized as “Normal.”

#### 3D Human Pose Estimation Using VideoPose3D

We used the VideoPose3D model [16] for estimating 3D human poses from single RGB images. Using a fully convolutional architecture with dilated temporal convolutions, VideoPose3D integrates 2D key points across multiple views and timeframes, enhancing the accuracy and robustness of 3D reconstructions by overcoming the limitations of single-view occlusions. In supervised settings, this model has outperformed previous benchmarks, achieving an 11% improvement in mean per-joint position error on the Human3.6M data set [33] showing significant advancements on the HumanEva-I data set [34]. Particularly in semisupervised scenarios with limited labeled data, VideoPose3D surpasses existing state-of-the-art methods.

#### 3D Coordinates System

**Figure 2:**
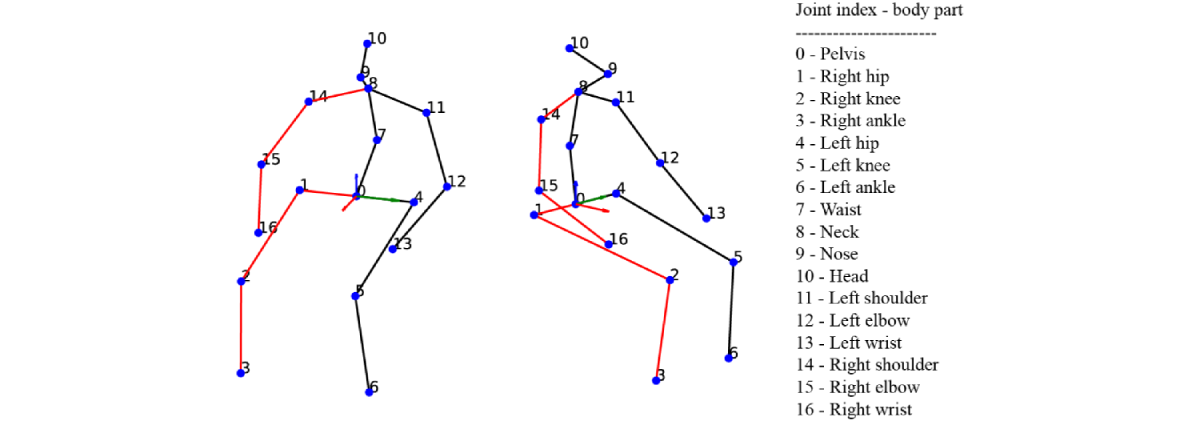
Skeletal model illustrating the 17 key anatomical points as identified by the VideoPose3D algorithm, numbered from 0 to 16 [16,35]. The red lines and dots denote key points on the right side of the body, highlighting the model’s ability to capture the complexity of human anatomy for pose estimation. Note that the skeleton is drawn such that the vector originating from the pelvis (#0) to the left hip (#4) is aligned with the y-axis of the coordinate system for visualization purposes.

VideoPose3D estimates 3D human joint key points, capturing kinematic information across 17 key points originally defined by the Human3.6M data set [33], as depicted in Figure 2 [16,35]. The 3D coordinates are defined along the x-, y-, and z-axes, with each coordinate value normalized within a range of –1.0 to 1.0. In this system, the x-axis corresponds to depth, the y-axis to width, and the z-axis to height as shown in Figure 2 [16,35].

We applied the normalization techniques for 3D human poses, thereby enhancing the model’s resilience to variations in viewing direction.

#### Performance Evaluation

We used test accuracy as a performance measure of the proposed recognition task. Accuracy is defined as the ratio of samples that the model predicts to correspond to the data labeling values among the total number of samples, which is expressed as follows:



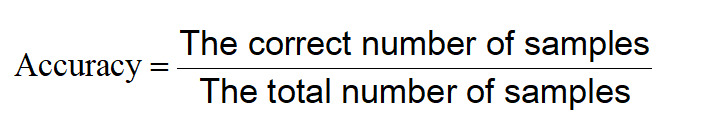



The *F*_1_-score is used to evaluate the classification performance of each class, which is defined as the harmonic average of the precision and recall, as follows:



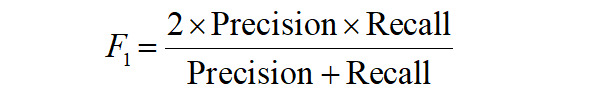



To evaluate all classes evenly, regardless of the proportion of each class c, we used the unweighted mean of per-class *F*_1_-scores, macro average, *F_m_*, which is defined by the following formula:



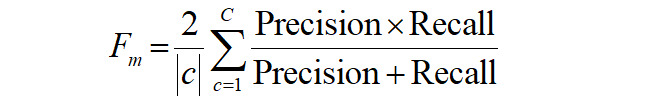



### Experiment

#### Data Collection and Preprocessing

For this study, we mainly used the publicly available StateFarm data set [17], which comprises images of 26 individuals. Each image has a resolution of 640 by 480 pixels and is in a 3-channel (RGB) format. The data set was originally curated to classify the level of drivers’ attention in 10 different scenarios: safe driving (c0), texting with the right hand (c1), talking on the phone with the right hand (c2), texting with the left hand (c3), talking on the phone with the left hand (c4), operating the radio (c5), drinking (c6), looking behind (c7), doing hair and makeup (c8), and talking to a passenger (c9). In our research, we have adapted this data set to assess the risk of FHP. To ensure the accuracy of our reclassification, we engaged in a detailed cross-validation process with the help of 2 physical therapists (Bo Yeon Park and Dong Hyeon Park, from the Department of Physical Therapy, Namseoul University, Cheonan, Korea). These experts, specializing in neurological rehabilitation, manual therapy, and pain management, practice at university hospitals, also known as tertiary medical institutions. Their expertise in posture assessment was crucial for identifying FHP, characterized by the contraction of the upper cervical region, leading to disrupted body alignment. To enrich our data set, we also incorporated additional images from a public pose repository [18] and extracted data sets from publicly accessible digital photos and videos including YouTube. This effort yielded a total of 2387 samples, with 1528 samples classified as “normal” and 859 samples classified as “FHP.” Specifically, we included 1220 “normal” samples and 310 “FHP” samples from the StateFarm data set [17].

To extract the 3D pose, we initially estimated 2D joint key points using Detectron2 [19]. Following this, we used Videopose3D [16] to estimate the 3D human pose. This 2-step process involves accurately detecting the position of key anatomical points on the human body in a 2D plane using Detectron2, a powerful tool for object detection and key point estimation. After obtaining these 2D key points, Videopose3D, a specialized model for 3D pose estimation from video data, is used to infer the 3D structure and position of the human body. In this study, the mean time taken to infer 2D joint key points using Detectron2 was 0.6170 (SD 0.0193) seconds for each image. Additionally, the process of inferring 3D joints from these 2D key points required an average of 44.1 (SD 3.35) milliseconds per sample.

#### Distribution of Rounded Shoulder Angles

Measuring the craniovertebral angle (CVA) is essential in detecting FHP. However, accurately gauging the 3D position of the C7 vertebra, a key component for calculating CVA, is challenging with current methodologies without dedicated equipment. This limitation restricts the practicality of such measurements in everyday settings.

**Figure 3:**
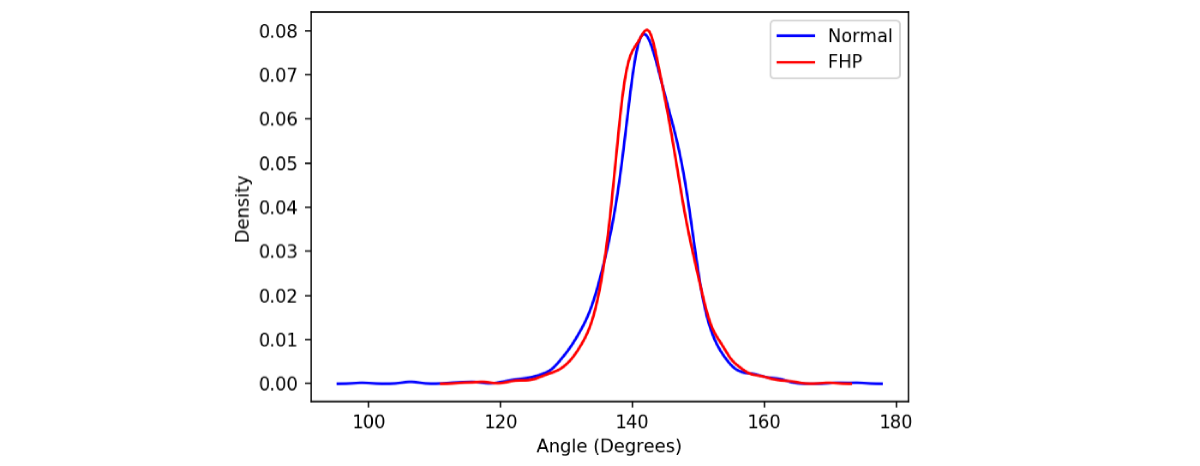
KDE plot illustrating the distribution of the shoulder angle for samples labeled as normal (blue line) and those labeled as FHP (red line) within the classified data set. The overlap in these distributions highlights the challenge of distinguishing between the 2 categories based solely on the shoulder angle, emphasizing the complexity involved in identifying FHP using only this measurement. FHP: forward head posture; KDE: kernel density estimation.

Additionally, 3D pose estimation models, such as Videopose3D, which estimates 3D human pose from a single 2D image, lack a direct method for estimating the CVA. This is because they are trained to identify general human anatomical landmarks, such as shoulders, elbows, and knees, without specifically accounting for the CVA. Therefore, rather than directly measuring the CVA, we initially calculated several feasible measures, including the shoulder angle, noting that FHP arises when spinal neutral alignment is compromised. To explore the patterns within the measured shoulder angles, we analyzed the distributions in the data set using kernel density estimation plots. These plots offer a visual interpretation of how the shoulder angles are distributed according to posture type—FHP or normal—as depicted in Figure 3.

**Figure 4:**
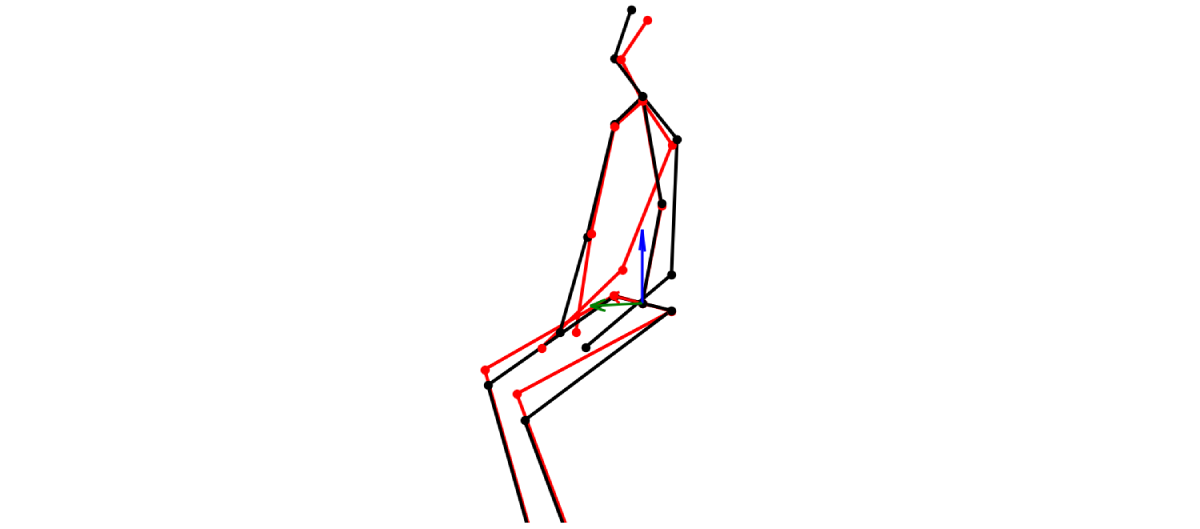
Visualization of 2 postures with their spines aligned for comparison. The posture indicative of FHP is depicted in red, whereas the normal posture is shown in black. This visual comparison highlights that in the FHP posture, the shoulders are rounded and shifted forward compared to the normal, more vertically aligned posture. FHP: forward head posture.

Contrary to our expectations, distinguishing FHP from normal posture solely based on these distributions proved to be nontrivial, as there is a significant overlap in the data as shown in Figure 3. This overlap suggests that while the shoulder angle is a relevant factor, it may not be sufficient on its own to identify FHP with high confidence. As depicted in Figure 4, which presents a comparison of 2 postures with their spines aligned for clearer visualization, it becomes evident that the use of shoulder angles alone for the detection of FHP is not sufficient. The figure underscores the complexity of posture analysis, suggesting the need for a more comprehensive feature set capable of capturing the subtleties of spinal alignment beyond the shoulder angle alone. Accordingly, we used a neural network as an advanced function approximator to effectively identify FHP using predicted 3D human poses.

#### Input Data

Considering that upper body joints remain visible and can be accurately estimated while sitting, we incorporated all upper body joints as inputs for our system. Furthermore, we explored various feature combinations to determine the optimal set for our objectives, as summarized in Table 1.

**Figure 5:**
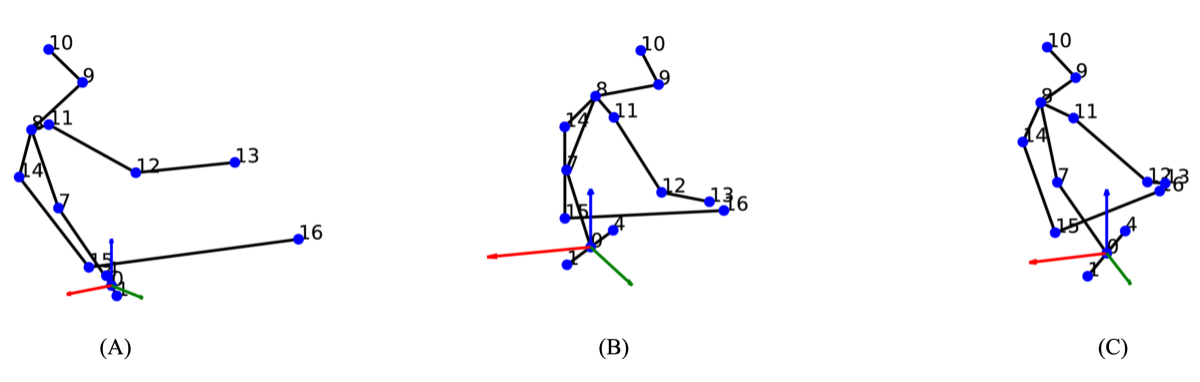
Illustrations of extracted upper-body poses for model training. (A) driving, (B) texting while walking, and (C) viewing a tablet screen.

Figure 5 presents examples of the upper-body poses used for training our model. Out of the 17 available joints, we selected 13 that represent the upper body, as the lower body is typically obscured during sedentary activities.

To account for the connectivity information between joints, such as from the pelvis to the left or right hip and spine joints but not from the pelvis to the right or left hand, we designed a graph-based neural network. This is further detailed in the following section.

**Table 1:** Features used in this study.

Data dimension	Input joints (original index)	Joint region
39	0, 1, 4, 7, 8, 9, 10, 11, 12, 13, 14, 15, 16	All upper body joints

#### Machine Learning Algorithm

**Figure 6:**
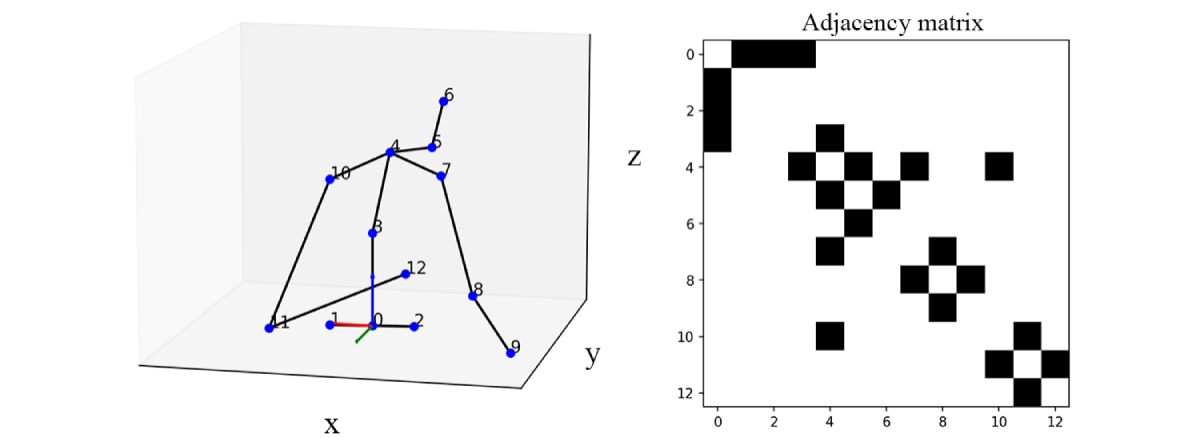
Adjacency matrix representing structural connections between human anatomical joints for FHP detection. Black squares denote direct connections used in the GCN, such as those from the pelvis to the hips and spine, indicated by the filled cells in the first row corresponding to the connections (0, 1), (0, 2), and (0, 3). Here, indices are reassigned from 0 to 12, differing from the original definition as shown in Figure 5. FHP: forward head posture; GCN: graph convolutional network.

For this study, we used a GCN, explicitly crafted to analyze the spatial configuration and alignment of the upper body’s anatomical key points in 3D space. This GCN aims to implicitly learn the intricate relationship between the estimated 3D key points and the correct posture, specifically to identify FHP. Figure 6 shows the adjacency matrix, which shows the structural connections between human anatomical joints used in this study. For example, the first line of the matrix can be translated into [0., 1., 1., 1., 0., 0., 0., 0., 0., 0., 0., 0., 0.], meaning the pelvis is connected to the left or right hips and spine.

The GCN used in this study consists of 2 graph convolutional layers, each followed by dropout for regularization. After graph convolutions, the high-level features are flattened and passed through a dense layer before the final classification layer, which outputs the probabilities for the given number of classes. This architecture is designed to exploit the graph structure data for classification tasks. To optimize our model, we conducted hyperparameter tuning for the dropout rate via GridSearchCV, ensuring optimal model performance. For training, we used a batch size of 16 to ensure efficient and effective learning. As a baseline algorithm, we adopted a conventional feedforward neural network (FFNN), a type of neural network architecture where information moves strictly in a forward direction—from the input nodes, through the hidden layers, to the output nodes. We trained our model using the Adam optimizer and categorical cross entropy as the loss function, which is appropriate for our class classification tasks where labels are provided in a one-hot encoded format. The model’s performance was evaluated based on its accuracy metric, allowing us to assess how well the model predicts the correct posture category for a given input image. For training, validating, and testing the model, we split the data using a 3-way split ratio of 70% (training), 15% (validation), and 15% (test).

## Results

**Figure 7:**
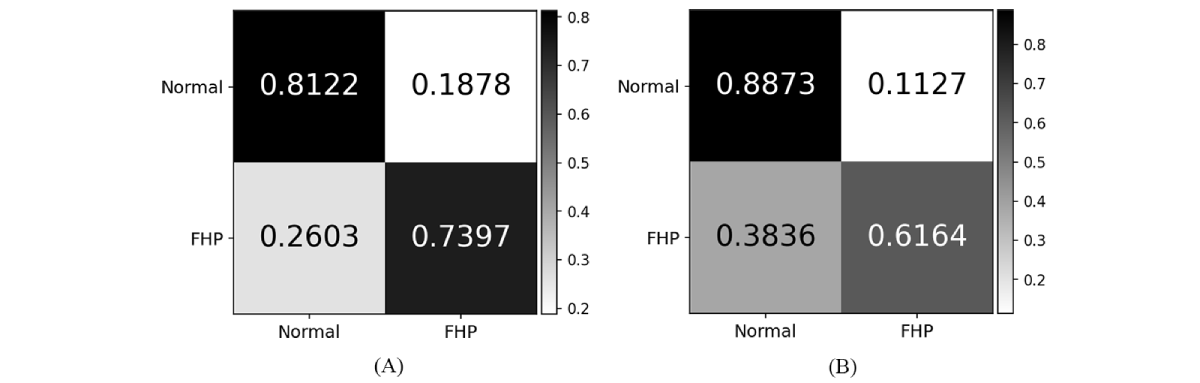
Confusion matrices for different training conditions. Matrix (A) shows the classification results for the proposed graph convolutional neural network. Matrix (B) presents the results from the baseline model for comparison.

Figure 7 shows the confusion matrices for the proposed GCN and the baseline model, while Table 2 provides more detailed classification results on the test data set.

**Figure 8:**
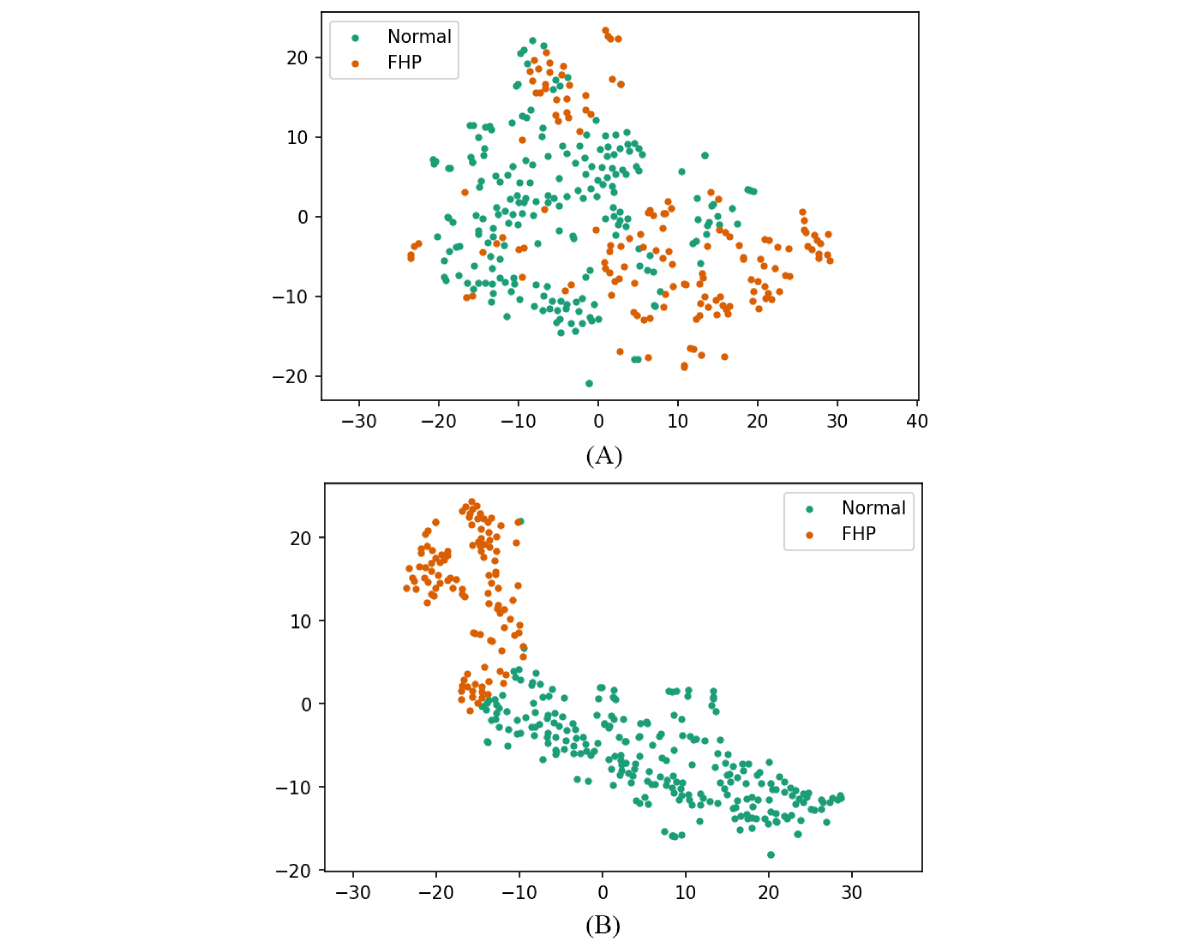
t-SNE plots of the test data set. Each point represents a 3D pose, colored according to its class as inferred by the model. Panel (A) presents the GCN model, which demonstrates more dispersed clustering. Panel (B) showcases the FFNN model, characterized by highly condensed clusters. This indicates that the GCN model is capturing a nuanced separation between classes, which could potentially generalize well to new, unseen data although the FFNN model shows clearer class separation to the given data. FFNN: feedforward neural network; t-SNE: t-distributed stochastic neighbor embedding.

We explored the high-dimensional feature space learned by our models using t-distributed stochastic neighbor embedding (t-SNE), a technique that reduces the dimensionality of data while preserving the relative distances between points [36]. In these t-SNE plots, each point corresponds to a 2D representation derived from the original 64-dimensional feature space, which is the output from the penultimate layer of our GCN and the FFNN, respectively. The process of t-SNE projection allows us to visualize the clustering and separation of data points, which indicates how well the network has learned to distinguish between different classes. t-SNE plots reveal the clustering and separation in the model’s learned feature space, indicating the network’s ability to discriminate between classes and its potential for generalization (Figure 8).

**Table 2:** Classification results on the test data set.

Metrics or model	GCN^a^ (%)	FFNN^b^ (%)
Precision (class 0)	81.99	77.14
Recall (class 0)	81.22	88.73
*F*_1_-score (class 0)	81.60	82.53
Precision (class 1)	72.97	78.95
Recall (class 1)	73.97	61.64
*F*_1_-score (class 1)	73.47	69.23
Overall accuracy	78.27	77.72
*F*_1_-score (macro, )	77.54	75.88

^a^GCN: graph convolutional network.

^b^FFNN: feedforward neural network.

## Discussion

### Classification Performance

The GCN model exhibits a more balanced performance between classes compared to the FFNN model. For class 0 (Normal), the GCN model achieves a precision of 81.99% and a recall of 81.22%, closely mirroring its performance for class 1 (FHP) with a precision of 72.97% and a recall of 73.97%. This balanced accuracy suggests the GCN model is effective in distinguishing both classes with similar proficiency.

In contrast, the FFNN model shows a higher disparity in its performance between the 2 classes. It has a higher precision (78.95%) for class 1 compared to the GCN model but at the cost of a significantly lower recall (61.64%), indicating it misses a considerable number of actual FHP cases. This may arise from the FFNN model’s tendency to make fewer false positive predictions at the expense of increased false negatives, suggesting it is more conservative in predicting FHP cases. For class 0, the FFNN model’s recall is higher (88.73%) than its precision, suggesting it is more reliable in identifying normal posture but with a tendency to incorrectly classify FHP as normal. In short, the FFNN model demonstrates a trade-off where it is more cautious and thus precise in identifying FHP cases but sacrifices the ability to recall all actual FHP instances. This results in more missed detections of FHP while being more accurate in confirming normal postures.

Overall, while the accuracy of both models is similar (GCN: 78.27%; FFNN: 77.72%), the GCN model offers a more balanced and equitable classification across classes, indicating a slightly better generalization ability to different types of postures. The FFNN model, although slightly lower in overall accuracy, demonstrates strength in identifying normal posture but struggles with reliably detecting FHP cases.

### Learned Feature Space

The t-SNE plot from the GCN model shows a more dispersed clustering, with the 2 classes (Normal and FHP) displaying some degree of overlap but also areas of distinct grouping. This suggests the GCN model is learning a nuanced separation between classes that could generalize well to unseen data.

In contrast, the t-SNE plot from the FFNN presents highly condensed clusters, indicating that while the model distinguishes very well between the 2 classes on the training data, it might be too tailored to the specifics of that data, potentially leading to overfitting.

From the comparison of the ratio of the first two eigenvalues between the two models, we can infer the degree of anisotropy present in the data. The GCN model, with a ratio of 0.4986, suggests a more isotropic distribution, indicating that the data points in high dimensional feature space spread more evenly across the principal components. In contrast, the FFNN model, with a significantly lower ratio of 0.2251, implies a more anisotropic distribution, where the learned features from the data are predominantly stretched along the first few principal components. This difference highlights the GCN model’s ability to capture a more balanced representation of the data, suggesting the potential for better generalization across varied data sets.

### Limitations and Future Work

This study introduced a novel approach to detect FHP by leveraging 3D human pose data extracted from single RGB images. While we successfully used pose estimation to determine the 3D positions of joints and used neural network analysis to distinguish between FHP and normal postures, our methodology faces certain limitations.

**Figure 9:**
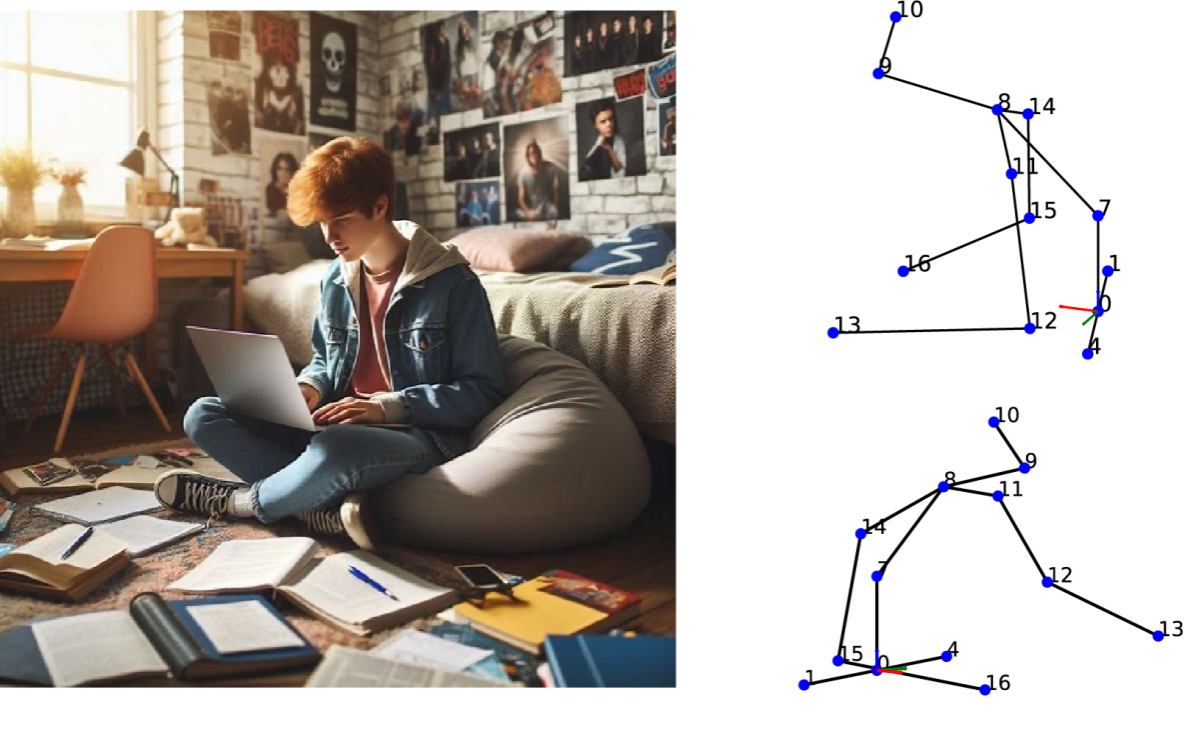
An example of pose estimation of an AI-generated image using Detectron2 and Videopose3D. This image was created using an AI-based image generation tool. The tool, powered by OpenAI’s DALL-E, uses text descriptions to produce detailed images. Note that the vector extending from the pelvis (#0) to the spine (#7) aligns with the z-axis in the coordinate system for visualization purposes. The broad use of AI-generated images to enhance our data set opens a promising path for future research, potentially providing a comprehensive solution to the current limitations. AI: artificial intelligence.

The validation of our model against a known data set may not fully encapsulate the wide range of postural variability present in real-world scenarios. This challenge is further exacerbated by potential biases within the data set and the lack of a comprehensive collection of diverse postures, limiting the model’s training scope. One approach to overcome this limitation involves the acquisition of a more varied set of bad postures. However, the ethical and practical challenges of encouraging participants to adopt incorrect postures intentionally make this solution less viable. An alternative strategy to enhance data set diversity without compromising ethical standards involves the use of artificial intelligence–generated images to simulate a wide range of bad postures. Such a technique would enable the expansion of our data set with postures that are underrepresented or particularly challenging to classify, thereby improving the model’s robustness and accuracy. Figure 9 demonstrates the extracted 2D and 3D human poses using Detectron2 [19] and Videopose3D [16], respectively. The extensive application of artificial intelligence–generated images to augment our data set presents a promising avenue for future research, potentially offering a comprehensive solution to the limitations currently faced.

In summary, while our current method marks a significant step forward in FHP detection, future work will focus on addressing these limitations by exploring innovative ways to enrich our data set and enhance model training. This will involve not only the inclusion of a wider array of postures but also the continued refinement of our neural network model to adapt to the nuanced complexities of human posture classification.

### Conclusions

FHP can result from various factors including misalignment of seating ergonomics, habitual body mechanics, prolonged use of electronic devices, or a general lack of postural awareness. The occurrence of FHP is notably prevalent during sedentary activities, highlighting the need for a practical system capable of detecting FHP to enhance postural awareness among users. Recognizing the challenges associated with obtaining precise measurements of the CVA—a crucial landmark for FHP diagnosis—without specialized equipment, this study aims to develop an accessible machine learning solution. We propose a neural network-based model that leverages the spatial relationships of 13 key upper body joints to accurately identify FHP. To validate our approach, we trained the model using data collected from multiple publicly available sources. Experimental results demonstrated that the GCN-based approach outperformed the baseline model in terms of FHP detection, particularly in achieving a higher recall for FHP cases. The t-SNE visualization for the GCN model reveals a broader dispersion of clusters, where the categories (Normal and FHP) exhibit overlapping to a certain extent alongside clearly segregated groupings, indicating that the GCN model is effectively distinguishing between the classes in a manner that may offer good generalizability to new, unseen data sets.

In future work, we aim to broaden the application of our approach to various real-world contexts, such as enhancing office ergonomics, where continuous monitoring of posture is crucial.

## Abbreviations

CVA: craniovertebral angle
FFNN: feedforward neural network
FHP: forward head posture
GCN: graph convolutional network
RGB: red, green, and blue
t-SNE: t-Distributed Stochastic Neighbor Embedding

## Data Availability

The data analyzed during this study are available in the StateFarm data set [17] and a public pose repository [18].
